# How the Perception of Public Official on Organizational Culture Influences Procedural Justice in Environmental Policy Processes

**DOI:** 10.3389/fpsyg.2021.626210

**Published:** 2021-09-27

**Authors:** Meng Yuan

**Affiliations:** School of Public Policy & Administration, Chongqing University, Chongqing, China

**Keywords:** organizational culture, culture theory, policy process, procedural justice, perception of public officials

## Abstract

How does the organizational culture of local governments influence the type and extent of procedural justice in environmental policy processes? Using the culture theory developed by Mary Douglas and others, this research seeks to bring a new conception and new measures of organizational culture to the study of policy making by local governments. To contribute to the development of the conceptualization and measurement of procedural justice in the environmental policy processes of those governments, item response theory (IRT) graded response model (GRM) is used to show variations in difficulties and frequencies of adopting distinctive public participation strategies for improving procedural justice across local governments. In this study, original survey data is collected from Illinois municipalities and a finding is suggestive of cultural variables explaining the two dimensions of procedural justice, equal and authentic public participation, while other variables can, at best, explain only the equal public participation. Furthermore, as hypothesized, egalitarianism increases both equal and authentic public participation, individualism increases equal public participation, and fatalism decreases both.

## Introduction

Public participation is central to the demands of procedural justice in environmental justice (EJ). However, there are large gaps between public participation as traditionally conceived and the ideal of procedural justice. In EJ research, procedural justice is defined as equal and authentic access to environmental policy-making processes (Hamilton, 1993; Bullard, 1994; Schlosberg, 2007; Walker, 2012). Relatedly, the US Environmental Protection Agency (EPA) emphasizes the population's *meaningful involvement*, including (1) the access of the population to the development of environmental policies regardless of race, color, national origin, or income and (2) the contributions of all participants in influencing policy decisions in the decision-making process (United States Environmental Protection Agency, 2016). Therefore, procedural justice is a more demanding concept compared to public participation (Hamilton, 1993, Gould, 1996; Lake, 1996; Young, 2002; Schlosberg, 2007) due to its insistence on equal and authentic public participation. The insistence on equal and authentic public participation in EJ movements is due to the crucial link between procedural justice and distributional (or substantive) justice in an environmental policy. As observed by EJ scholars, the disproportional exposure to environmental toxins or inequitable distribution of environmental goods reflects a general lack of participation and the influence of people of color or lower-income groups in environmental policy processes (Hamilton, 1993, Lake, 1996; Schlosberg, 2007). As a result, being distinct from traditional public participation, procedural justice has the normative values of caring for disadvantaged and marginalized groups and empowering them. More specifically, on the dimension of equal public participation, this study asked to what extent local governments select the three types of public participants, including lay citizens, randomly selected participants, and underrepresented groups, in environmental policy processes. On the dimension of authentic public participation, this research asked to what extent local governments adopt three strategies of delegating the power to the public, including consulting with the public, co-governing with the public, and delegating direct power to the public in final decisions, in environmental policy processes.

To understand why some local governments consider procedural justice more seriously than others regarding environmental policy processes, EJ scholars have conducted a variety of case studies (Finn and McCormick, 2011; Schrock et al., 2015). Most of them mainly focused on external factors and political pressures faced by local governments such as racial diversity, socio-economic conditions, and community-based partnerships(Freudenberg, 2004; Di Chiro, 2008; Faber and McCarthy, 2012; Schrock et al., 2015) but overlooked the policy supplier of local governments in the political market. As a result, a few EJ scholars have started to notice the role of political cultures and values within the local governments themselves in explaining the municipal efforts to EJ (Finn and McCormick, 2011). However, to the best of my knowledge, they have not developed empirical studies to specify or quantify the cultures and values within the government that influence procedural justice.

Cultural theory (CT), developed by Mary Douglas and others (Douglas and Wildavsky, 1982; Schwarz and Thompson, 1990; Thompson et al., 1990; Wildavsky, 2006), conceptualizes and derives the political cultures of egalitarianism, hierarchy, individualism, and fatalism from the two dimensions of social and political relations, which have been used to study organizational culture and its influences (Hood, 1998; Maesschalck, 2004; Bellamy et al., 2008; Lodge and Wegrich, 2011; Swedlow, 2014; Matheson, 2017). Although CT has been used to study the choices of citizens in public participation in policy processes (Gastil, 2000; Hoppe, 2010; Ney and Verweij, 2014; Trousset et al., 2015; Linsley et al., 2016; Saab et al., 2017), the only research by Smith-Walter (2015) has used CT to study the choices of governments in public participation in environmental policy processes. In his research, Smith-Walter studied how the cultural worldviews of transportation planning staff influence the differences in their preferences for three public participation mechanisms. However, his research was to operationalize CT only as individual-level worldviews rather than as organizational culture, which is the focus of this study. It is argued that measuring organizational culture is important for the case of two reasons. First, people may have different cultural biases dominating the different parts of their lives (Thompson et al., 1990). Operationalizing CT without accounting for institutional contexts and attachments can lead to uninterpretable results. Second, institutions are the core of power and thus likely to influence policymakers, so it is important to measure institutional influences in addition to the cultural biases of individuals.

To fill the abovementioned research gaps, this research uses CT to answer: (1) How are the commitments of local governments to procedural justice manifested in the two dimensions of procedural justice: equal public participation and authentic public participation? Specially, this article uses the polytomous item response theory (IRT)graded response model (GRM)—weighing different levels of specific public participation strategies on these two dimensions, the acts of recruiting lay citizens vs. the recruitment of underrepresented groups, for example, (2) how well organizational culture explains these two dimensions of procedural justice. To test the hypotheses regarding the relationship between the organizational culture of a local government and its commitment to the two dimensions of procedural justice, the statistical analyses were conducted in two steps. The first step would mainly consist of a series of non-parametric analyses of the mean of procedural justice across the four types of dominant organizational culture. The dominant culture is generated by selecting the highest average value of a cultural type on which the respondent scored the highest. In the second step, four cultural indices, including egalitarianism, individualism, hierarchy, and fatalism, were used in bivariate and multivariate regressions for more definitive testing of the aforementioned hypotheses.

## Public Participation and Procedural Justice in Environmental Policy Processes

Much literature has attempted to define the term public participation. The best-known study is Arnstein (1969) ladder of public participation and subsequent research reports the increasing influence of public participation on policy decisions (Shannon, 1990; Moote et al., 1997). Ideally, public participation requires that public interest must be reflected in final decisions (Cvetkovich and Earle, 1992; Goodin, 1993; Hampton, 1999). Another way to define public participation is to define the scope of the public or who is involved in the policy process (Moote et al., 1997; Blake, 1999). For example, the term “public” is distinct but can include “citizens,” (i.e., eligible voters) “community,” (i.e., members of a neighborhood or area) and “residents.” (i.e., inhabitants of a particular locale) It is also distinct from the organized groups that have vested interests in an issue but do not necessarily include the members of the lay public (Nabatchi and Amsler, 2014). However, the other dimensions of public participation, such as at what point the public is involved, the communicative method used, and the purpose of public participation, have also been discussed (Fung, 2006; National Research Council, 2008).

Public participation is a key demand for procedural justice. However, procedural justice in EJ movements is a concept distinct from the overall public participation in its focus on equal and authentic public participation (Gould, 1996; Lake, 1996; Young, 2002). More specifically, in environmental policy processes, equal participation requires not only the participation of the public but also ensures that marginalized and disadvantaged groups have a place at the table (Sarokin and Schulkin, 1994; Eden, 1996; Corburn, 2005; Fortmann, 2008; Shilling et al., 2009). Authentic participation requires that public input is taken seriously by authorities and is influential in final decisions (Cvetkovich and Earle, 1992; Hampton, 1999; Schlosberg, 2007). Putting these elements together, procedural justice in environmental policy processes should be defined as a process in which all people, regardless of race, ethnicity, income, national origin, or educational level, can equally participate and have a meaningful influence in environmental policymaking (Schlosberg, 2007; for related definitions, see Walker, 2012). Therefore, this study focused on the two dimensions of procedural justice: equal and authentic public participation.

### Dimension 1: Equal Public Participation

This dimension examines the extent to which local governments select the members of the public to participate in environmental policy processes (Rowe and Frewer, 2005). This research borrowed the three categories of public participants as defined by Fung (2006):

- *Targeting underrepresented groups*: institutions of open participation with incentives for the targeted underrepresented or inactive groups to participate are the most intensive strategy for political equalization (Lukensmeyer and Brigham, 2002; Fung, 2006). In participation mechanisms such as referenda, public opinion surveys, consensus conferences, and focus groups, the underrepresented groups can be invited. Moreover, in practice, the participation mechanism can be more successful in achieving the goal of high representation when local governments use better public outreach and optimize the accessibility of the process to underrepresented groups and communities (Bryson and Kathryn, 2012).
- *Randomly selecting participants:* employing a random selection can promote a representation by improving descriptive representativeness (Fung, 2006), meaning that the representatives mirror the larger populations in key demographic characteristics such as race, gender, ethnicity, or religion. A local government may also select a random stratified sample of the affected population (Rowe and Frewer, 2013). Examples of participation mechanisms include the following: deliberative polling (Fishkin, 1995), citizens jury/panels (Smith and Wales, 2000), and planning cells (Dienel, 1999).
- *Participation of lay citizens*: these are unpaid citizens willing to invest substantial time and energy to act as representatives. Lay citizen involvement enhances equal participation if the stakeholders are properly utilized because they can serve those who have similar interests or perspectives but choose not to participate (Fung, 2006). Therefore, the participants participate voluntarily and are not passively invited by the government. Moreover, they participate as an individual resident, not as a member of any given interest group. Participation mechanisms for lay citizens include but are not limited to Citizens Advisory Committees, Neighborhood Association Boards, and Negotiated Rulemaking processes.

### Dimension 2: Authentic Public Participation

This dimension asks to what extent the public delegates the power in environmental policy processes. The power dimension includes three strategies, which reflected public interests in final policy decisions.

- *Direct power*: it can promote justice as the highest level of empowerment it addresses the structures of corruption and exclusion that generate benefits for the advantaged; the recommendations offered by merely advisory mechanisms are typically ignored. An example of direct authority is “Open” Town Meetings (Fung, 2006), which enable residents to directly deliberate and vote on laws and budgets (Zimmerman, 1999). Other examples are referenda and negotiated rulemaking (Rowe and Frewer, 2013).
- *Co-governing*: in some venues, the public may participate in a kind of co-governing partnership with local government in which they collaborate with public officials to make plans or develop strategies for public action (Fung, 2006). Despite allowing less power, a co-governing partnership is also a mechanism through which the general public can exercise direct power in policy-making processes (Fung and Wright, 2003), for example, Citizens Juries (Rowe and Frewer, 2013; the United States Environmental Protection Agency, 2016) and Citizen Advisory Committees and Public Task Forces (International Association for Public Participation (IAP2), 2018).
- *Consulting*: public officials may preserve authorities and powers in final decision-making in public participation mechanisms, such as public hearings, public comment devices, and focus groups. However, rather than just informing the public when the decision has been made, public officials can commit themselves to obtain the inputs from the public and take account of these inputs when the final decision is made (Fung, 2006).

## CT in Government Studies

Cultural theory has been used to study the policy process preferences or public participation in governmental policy processes, but in general, this research focuses on the attitudes and behaviors of the general public (Trousset et al., 2015; Linsley et al., 2016; Zanocco and Jones, 2018). There are also a few examples of CT research analyzing collaboration in and around the government (Weare et al., 2014; Conner et al., 2016). In 2014, Ney and Verweij (2014) combined Fung and Wright (2001) eight institutional designs of public participation and the four types of culture in CT to categorize institutional design choices for public participation into a 4 × 8 matrix. However, they did not go so far as to map mechanisms in a manner that made hypothesis generation and statistical testing possible (Smith-Walter, 2015). Smith-Walter (2015) is the only research to date to empirically investigate the relationship between CT worldviews of administrators and public participation designs in public planning. However, because of his focus on the CT worldview of planners, his research fails to capture the role of organizational culture, and his operationalization and measurement of culture are not consistent with the stress on public management styles (Hood, 1995, 1998). Therefore, investigating organizational cultures is vital for gaining a full picture of the influence of culture on public participation designs.

In government studies, many are familiar with Elazar's (1970) conception of individualistic, moralistic, and traditionalistic political cultures, which has overlapped with the cultures defined by CT. Elazar's political culture was used to explain how state and local politics function and how political culture relates to the other factors that affect the outcomes in state and local politics including political participation at the state level (Sharkansky, 1969; Johnson, 1976; Morgan and Watson, 1991). Related research found that popular participation is encouraged by the state in moralistic cultures and discouraged by the state in traditionalistic cultures (Sharkansky, 1969; Johnson, 1976). The state in individualistic political culture either falls in the middle when a unidimensional scale ranging from moralism through individualism to traditionalism is used to measure the three political subcultures (Sharkansky, 1969; Morgan and Watson, 1991).

Elazar's conception of political culture has various limitations such as an inductively generated typology of cultures that are arguably overcome by CT. CT is a theory rather than an *ad hoc* inductively generated set of political-cultural labels. CT has developed two theoretical propositions. First, the “requisite variety condition” states that the four cultures need one another to be viable (Thompson et al., 1990, p. 2) and thus the elements of all four cultures should be expected to be present within the public management system. However, Elazar's political-cultural types are neither mutually exclusive nor jointly exhaustive (Thompson et al., 1990). For example, moralistic culture tends to run together with the two distinct ways of life: egalitarianism and hierarchy. Encouraged by Hood's (1998) application of CT to explain the regulatory styles of public organizations, many public administration scholars use CT as an organizational theory and find that CT adequately accounts for various forms of control over bureaucracies (Hood, 1995, 1998; Bellamy et al., 2008; Lodge and Wegrich, 2011; Swedlow, 2011; Wouters and Maesschalck, 2014; Perri, 2014; Matheson, 2017). For example, CT scholars increasingly challenge the approach of leaving out fatalism and provided evidence that the four cultures can explain the varieties of regulatory approaches (Hood, 1998; Lodge, 2011; Lodge and Wegrich, 2011; Nakamura, 2016). Fatalism is partly an unintentional consequence of the strong regulation of government, the increasing complexity in the roles of public administrators, the shift of governance toward privatization, and quasi-privatization (Dunleavy, 1989; Hood, 1998; Ney and Verweij, 2014) since New Public Management. Similarly, public participation research recognized their failure to capture the cultural components in the network organizations that can facilitate the efforts of local governments in public participation (Yang, 2005). Second, the “compatibility proposition” (Thompson et al., 1990, p. 2) states that the four cultures shape the specific values/beliefs and behaviors of individuals in social contexts such as organizations to maintain the viability of the system. However, as applied by him in his seminal Cities of the Prairie (1970) and subsequent work, Elazar's conception of political culture is considered as affiliations to religious groups or as intertwined with individual interest and social-economic status, rather than originating in social and political relations, which are the core of power relations, a central concept in political science (McClurg and Young, 2011, p. 39; Favre et al., 2019). CT conceptualizes and derives the political cultures of egalitarianism, hierarchy, individualism, and fatalism from the two dimensions of social and political relations. In other words, CT systematically assimilates the different forms of relations between public management organizations and the public into a coherent framework (Hood, 1998). Therefore, CT adds the theoretical and conceptual value beyond Elazar's cultural typology to procedural justice research and to understanding the multi-actor planning processes typically found in environmental policy processes.

## Theoretical Framework and Hypotheses

**Figure 1:**
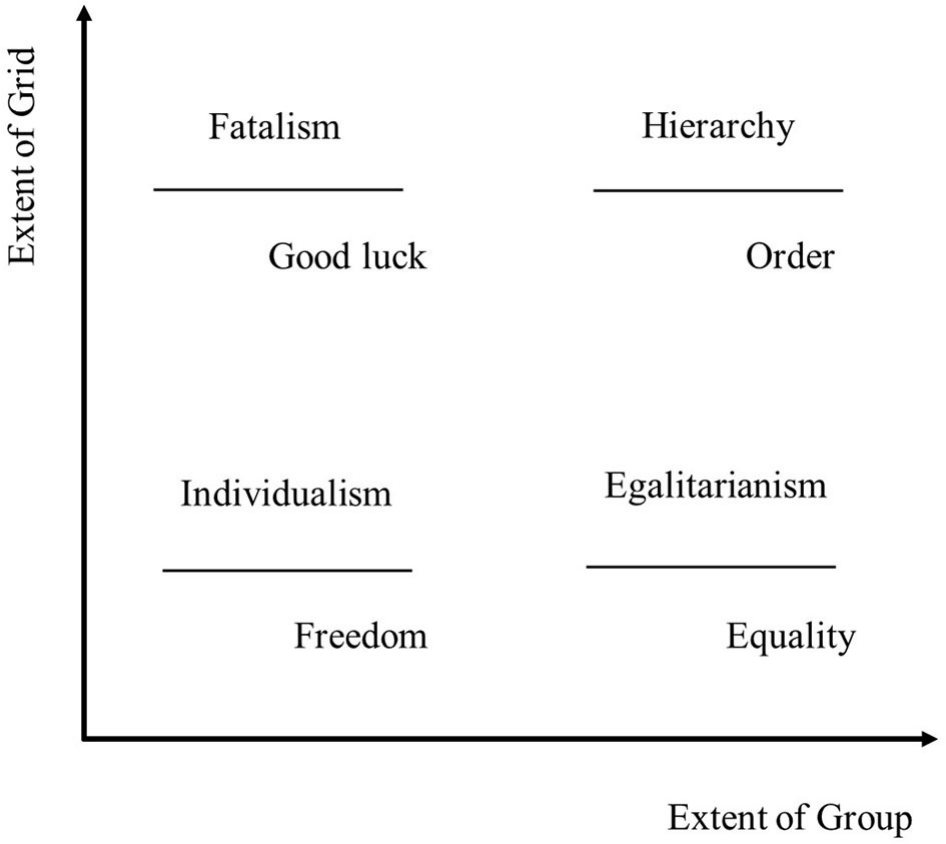
Grid-group culture theory.

In CT, culture is derived from a set of social relations that are categorized along the two dimensions: group and grid. These two social dimensions represent the most fundamental factors for controlling modern government (Hood, 1998; Nakamura, 2016). Group measures the extent to which officials share highly integrated patterns of thought and behavior, simultaneously, grid measures the extent to which government activities are constrained by rules and regulations (Hood, 1998; Entwistle et al., 2016; Nakamura, 2016). These dimensions and the resulting four patterns of social relations are depicted in Figure 1 (also see Swedlow, 2014): hierarchy, individualism, egalitarianism, and fatalism.

A cultural theory provides not just a typology of cultural types but offers a distinctive causal explanation of how cultural types derived from the theory cultivate particular packages of cultural biases that sustain those institutions while dynamically undermining others (Perri and Swedlow, 2016). CT suggests the functionalism among political or social relations, core values, and associated cultural biases within the four ways of life or cultures: each of these four patterns of social and political relations is justified by and, in turn, justify (and make plausible) particular kinds of cultural biases that refer to shared values and beliefs (Thompson et al., 1990; Hood, 1998; Wildavsky, 2006; Swedlow, 2014). As Swedlow (2011) suggests, “the social and political relations of the four ways of life specified by CT are simultaneously the specifications of the four ways of making decisions, constituting authority, and exercising power.” (p. 705).

Egalitarianism is organized as mutuality, which involves weak formal leadership and relies heavily on communal participative decision-making involvement (Hood, 1998). Within organizations, the structures of reciprocity and group interaction are often considered as a viable alternative to a purely hierarchical style of management in which the essence of the organization is conceived akin to a principal-agent dilemma and control from the principals is needed (Hood, 1998). People in egalitarian relations value equality over liberty and order (Coyle, 1994; Swedlow, 2008, 2014). They suggest that “no one is prevented from participation in any social role because he or she is the wrong sex, or is too old, or does not have the right family connections.” (Rayner, 1992, p. 87) These beliefs are compatible with the central conception of procedural justice, equal participation. Therefore, egalitarian public officials are likely to adopt strategies to include marginalized groups and create a more inclusive policy process.

Moreover, a key doctrine for egalitarian public management is the idea of citizen coproduction in public service provision, with local communities being given a central role in oversight (Hood, 1998; Simmons, 2016). Public officials in egalitarian relations distrust professionalism in service production (Hood, 1998) and tend to be sensitive to risks that arise from the concentrations of power in the policy processes that may oppress others. They suggest that the authority should be vested within the community rather than in outside experts or institutionally defined leaders (Jenkins-Smith et al., 2014). These beliefs match with another central conception of procedural justice, authentic participation in which the general public should have direct power to influence policy decisions. Therefore, as expected hypotheses are stated as follows.

*Hypotheses 1a: The increase in egalitarian culture will increase the local governments' commitment to equal public participation*.
*Hypotheses 1b: The increase in egalitarian culture will increase the local governments' commitment to authentic public participation*.

Hierarchy is reflected in a structure of organizations that are socially coherent and operate according to the well-understood rules of procedure (Hood, 1998; Matheson, 2017). In hierarchical organizations, control implies a ladder of clarified authority, conscious oversight, and inspection. In addition, formal power is needed to approve or reject, to pronounce on disputes or complaints, to forbid, command, permit, and punish. Relatedly, hierarchical collectives are characterized by a marked boundary that distinguishes among the members of a group, further grouping them within the group in contrast to the lack of intraorganizational boundaries among egalitarians (Swedlow, 2017). Much of public management consists precisely of wielding authority over citizens and the government using some forms of oversight to steer the society (Hood, 1998).

Generally, public officials in a hierarchical culture value order over equality and liberty (Coyle, 1994; Swedlow, 2008). Unlike an egalitarian culture, public officials in hierarchical organizations will tend to consider that society and organizations should and need to be directed by the appropriate authority (Hood, 1998). Hierarchs suggest that nature can be controlled, but in doing so, individuals must be bound by tight societal prescriptions where experts manage their sphere (Leiserowitz, 2006; Jones, 2011). Therefore, they prefer a science- or an expert-dominance approach to solve environmental problems within the government. Consequently, this culture should leave little or no room for public participation. Consistent with their values, hierarchs will define their role as educating the public because “people have no inherent proclivity to goodness, so institutions and norms need to be designed and enforced to make them so.” (Jenkins-Smith et al., 2014, p. 494; Thompson et al., 1990) Hierarchical organizations distrust and devalue the participation of the public and avoid conferring power inclusive of public involvement. Therefore, as expected the hypotheses are stated as follows:

*Hypotheses 2a: The increase in hierarchical culture will decrease the local governments' commitment to equal public participation*.
*Hypotheses 2b: The increase in hierarchical culture will decrease the local governments' commitment to authentic public participation*.

Individualism is organized in quasi-market competition, antipathy to collectivism, and a preference for handling transactions by trading or negotiation rather than by the preset rules. In organizations, public officials engage in a market exchange with their employer when selling their labor (Matheson, 2017). Competition among officials for the promotion in the upper reaches of public bureaucracies is viewed as a more effective way to ensure responsiveness to the needs of the governments than the sanction of dismissal (Horn, 1995). Moreover, governments control clients by using competition among clients for government recognition and/or the allocation of time or facilities (Hood, 1998).

The influence of an individualistic culture on equal participation may be ambiguous. On one hand, for individualists, unnecessary processes can be more readily identified and removed when fewer incompatible objectives are included in the policy process. Equally, the more control emphasizes the output over a process or input, the more unambiguous the waste-finding process can be (Hood, 1998). Therefore, individualists are sensitive to the risks of delaying the planning process and costs and timewasting in building a consensus in public participation. Similarly, some scholars argue that individualists are skeptical of vesting too much power in the general public as it would represent imposing a decision made by self-interested participants as part of the public on considering the public as a whole (Smith-Walter, 2015).

However, on the other hand, because individualists care more about the efficiency of policy outcomes than the policy process, they may have no interest in deliberately excluding and disempowering any individual participant as long as their self-interests or rewards are guaranteed. Furthermore, because individualists value the freedom of individuals over other values such as order, local officers under this culture will support pluralist democracy with checks and balances to maximize individual freedom (Verweij et al., 2011) by providing equal political opportunities for the public to participate in a policy process. Similarly, although, for individualists, delegating direct authority over policy decisions to the general public may result in undesirable policy outcomes, they construct public service users as rational customers and suggest that the role of public managers is to match supply to demands of individuals and respond directly to feedback from them (Simmons, 2016). Therefore, individualistic local governments may also tend to provide some room for consulting with the public and establishing a co-governing partnership with the public. Therefore, as expected the hypotheses are stated as follows:

*Hypotheses 3a: The increase in individualist culture will increase the local governments' commitment to equal public participation*.
*Hypotheses 3b: The increase in individualist culture will increase the local governments' commitment to authentic public participation*.

Fatalism occurs where people have little control over their lives and are socially isolated, and simultaneously they are strongly bounded by rules and constraints (Matheson, 2017). Under this culture, public officials are struggling to maintain their positions in public organizations but are unable to use authority to do so (Perri, 2014). They will exhibit apathy, cynicism, and hopelessness (Matheson, 2017) and have no incentive to work hard or effectively because there is no reliable link between public preferences and their performance (Hood, 1998; Matheson, 2017). Public officials in this culture reject any form of participation and collective action because they suggest that the effects of cooperation are likely to be uncertain and problematic (Stoker, 2002). Similarly, they reject any check or intervention from the general public. Therefore, their policy goals are less likely to be achieved through collective action.

There is a possibility that fatalism might link to contrived randomness (Hood, 1998) in management, as distinct from a view of the world as ineluctably ruled by the fickle goddess of fortune. According to Hood, contrived randomness appears to be a reasoned response to the world perceived to be characterized by capricious, weak-tied, low-trust relations, and modest prospects for effective coordination (Stoker, 2002). Stoker (2002) further explains that the randomness strategy being deliberate is crucial and the word random implies that each member of the targeted population has an equal chance of being selected. Therefore, in the policy-making process, local officials in fatalism may also prefer randomly selected participants. However, it is somewhat in conflict with the fact that fatalistic relations are characterized as the denial of the possibility of coordination or collective action by Wildavsky who introduced and developed CT in the USA (Ripberger et al., 2014; Swedlow, 2014). With little agreement in the literature on the managerial response to fatalist conditions and on what a fatalist perspective on policy (or policy process) is, this research will leave this debate to empirical tests. As expected, hypotheses are stated as follows:

*Hypotheses 4a: The increase in fatalist culture will decrease the local governments' commitment to equal public participation*.
*Hypotheses 4b: The increase in fatalist culture will decrease the local governments' commitment to authentic public participation*.

## Materials and Methods

### Unit of Analysis

In this study, the unit of analysis is local governments within Illinois. Illinois is chosen for two reasons: first, Illinois is often considered a microcosm of the nation, including the dominance of individualism within it (Elazar, 1970; Dran et al., 1991). However, because of the physiographic divisions of the state, it combines within its boundaries much of the cultural, social, economic, historic, and geographic diversity of the USA (Elazar, 1970; Dran et al., 1991; Nowlan et al., 2010).

Second, Illinois might be a case where we can observe how a fatalistic culture is manifested in public management. Scholars observing the culture of Illinois and CT scholars alike have noticed that administrations beginning with strongly individualistic ordering may transform to fatalistic culture in response to the accumulated adversities that erode the capacities for negotiation and patron-client claque relationships (6, 2016). Therefore, Illinois, with its varied political cultures, including fatalistic culture, is a good state for an initial study of the influence organizational political culture as conceived in CT may have on public participation.

### Data Source

The survey designed to collect the data for testing the foregoing hypotheses was created and administered using the Qualtrics online survey software. Questions were designed to identify five components of commitment of local governments to procedural justice, organizational cultures, and some of the control variables that have been found to influence the commitment of local governments to procedural justice in sustainability plans (see the Appendix). This study uses another data source, the US Census of 2010, to identify other control variables such as communities' SES, racial diversity, and populations.

The survey was intended to be administered to individuals who are likely to be the most knowledgeable in local governments about public participation processes in sustainability policy. Therefore, the survey was sent to a sustainability manager or person with a similar position. If the city did not have such a person, the survey was sent to the city manager or chief administrative officer. In the survey recruitment email, the respondents were also asked to identify the person who is more appropriate if they feel that they are not the right person to answer the survey.

This ongoing survey was sent to 207 cities in Illinois with populations over 10,000 starting on April 2, 2019. Another round of the survey was conducted on 269 cities with populations from 2,000 to 10,000 starting on September 20, 2019. To increase the response rate, three reminder emails were sent out every 2 weeks after the original email had been sent. By July 2, 2019, 54 valid responses from larger municipalities were received. By November 19, 2019, 42 valid responses from smaller municipalities were received. As a result, the sample in this study for the current analysis consists of 96 (response rate: 20%) responses of local governments in Illinois.

Municipal characteristics for the overall sample are summarized in Table 1. Chi-square goodness-of-fit test is used to assess the sample representativeness. No significant difference in median household income was found between responded governments and sampling frame. Council-manager cities and cities with a population over 10,000 are overrepresented while village or towns and cities with a population size from 2,000 to 10,000 are underrepresented. It can be a result from the fact that sustainability policies are more likely adopted by cities with more populations. Moreover, council-manager cities hire city managers who are more responsible for and have a greater interest in cooperating with research on public participation.

**Table 1:** Demographics of the responding local governments (*n* = 96).

**Categories**	**R (*n* = 96)**	**SF (*n* = 476)**	**Residuals for** **Goodness-of-fit test**
Population			
Over 10,000	56%	43%	1.98
2000–10,000	44%	57%	−1.72
Form of government			
Mayor-council city	33%	33%	0.06
Council-manager city	37%	18%	4.26
Village or towns	30%	49%	−2.63
Median household income			
<59,200	31%	34%	−0.30
Over 59,200	69%	66%	0.21

*R, Respondents; SF, Sampling frame.*

*59,200 is the median level of median household income in Illinois*.

### Variables

#### Dependent Variable: Equal Public Participation and Authentic Public Participation Index

Two dependent variables are used for investigating the two dimensions of procedural justice: equal and authentic public participation. Additive indices of public participation strategies have the disadvantage of equally weighing different levels of commitment to specific strategies. For example, the act of recruiting lay citizens vs. recruiting underrepresented groups; consulting with the public vs. delegating direct power to the public. Therefore, this research uses IRT GRMs as the method for addressing this limitation: an index of adopted public participation strategies with weights that reflect (1) the difficulty of being adopted (e.g., how a few municipalities adopt them) with a specific level (e.g., never adopted, rarely adopted, and always adopted) and (2) how well each strategy discriminates between two municipalities, was created separately for equal and authentic public participation. In the IRT model, this index is known as a predicted latent trait or Theta “θ.”

More specifically, the equal public participation index includes the three survey items with ordered polytomous responses: lay citizens, randomly selected participants, and unrepresented groups. For each survey item, respondents will be asked to rate separately, using a five-point scale (1 = never to 5 = always), the frequency with which their local government included each of the participants: “lay, unpaid citizen stakeholders;” “randomly selected participants;” and “selectively recruited participants from the subgroups that are less likely to engage.” The authentic public participation index includes the three survey items with ordered polytomous responses: consulting, co-governing, and direct power delegated to the public. The respondents will be asked to rate separately, using a five-point scale (1 = never to 5 = always), the frequency with which public participation “provided consultation and advice for environmental policy process,” “co-governed with the local government to make environmental plans and policies or strategies,” and “exercised direct authority and power in the environmental policy process.”

#### Independent Variables: Organizational Cultural Indices

Two approaches are generally used by CT scholars to measure culture. One relies on the quadrants of the cultural typology (Jones, 2011; Liu, 2019) and is often used for measuring the dominant or strongest culture. In this approach, the scores on each item measuring each of the four cultural types are typically summed, and respondents are then assigned to the culture on which they score the highest. However, no culture exists in a vacuum, and any organization can be viewed as a hybrid of more than one culture (Wildavsky, 2006). Therefore, four cultural indices are used to measure the affinity of an organization with each of the cultures rather than assigning individual organizations to a unique culture.

In this study, respondents were asked to evaluate 12 randomly ordered CT questions (refer to the Appendix). These 12 questions are adapted from the three items with the highest loading factor in Wouters and Maesschalck (2014) research and correspond to one of the four CT types of culture. The respondents are asked with each question to place themselves on a scale from one to five, where one means strongly disagree and five means strongly agree. As a result, four organizational cultural indices, including egalitarian, hierarchical, individualist, and fatalist index, were created by calculating the mean of scores of each of the four organizational cultures.

#### Control Variables

In this study, two sets of control variables developed by existing research are used. The first set of variables specifies municipal factors including SES of the municipalities measured by the percentage of people with bachelor's degree or above and median household income, racial diversity measured by the percentage of whites, and population. The second set of variables specifies demographic factors of the local officers including gender (1: male; 0: female), race (1: white; 0: non-white), political ideology (from 1: strongly liberal to 5: strongly conservative), education (1: less than college; 2: 4 years college; 3: master; 4: Ph. D. or professional degree), and years of working in government (1: 1–3 years; 2: 3–5 years: 3: 5–8 years; 4: 8–10 years; and 5: more than 10 years). In all the models, continuous variables are standardized to compare the effects and make the interpretation easier.

### Data Analysis Process

Little's test indicates the missing values for organizational culture completely at random (*p* > 0.05). Therefore, if the number of missing data was 1, the average value of the other items measuring that construct was assigned. If the number was 2 or more, the data were recorded as missing. Cronbach's alpha was calculated to test the internal consistency of measuring organizational culture.

In this study, analyses of the relationship between organizational culture and procedural justice were conducted in two steps. The first would consist of one-way ANOVA tests of the mean of equal public participation index and authentic public participation index across the four dominant cultures. The dominant culture is generated by selecting the highest average value of cultural type on which the respondent scored the highest and is measured by dichotomous cultural variables: dominant egalitarianism, dominant hierarchy, dominant individualism, and dominant fatalism. In the second step, the four cultural indices were used in bivariate and multivariate analyses for more definitive testing of the hypotheses. A correlation matrix was produced. Moderate correlations have been found among the four cultural indices (*r* = 0.30–0.50), and a high correlation between the egalitarian and fatalist index (*r* = −0.62) has been found, which is theoretically reasonable given that the four cultures are derived from the two shared dimensions, grid and group, of political relations in CT. Therefore, each cultural index was included separately in multivariate analyses to prevent multicollinearity. Moreover, no correlation coefficient had a value of more than 0.40 among other variables, variance inflation factor (VIF) also found that VIF values for other variables are <2. Therefore, there is no multicollinearity for other variables in the models. Ordinary least squares regressions were run for each of the two dimensions of procedural justice: equal and authentic public participation.

## Results

### Step 1: Descriptive Analyses and One-Way ANOVA Tests

Table 2 summarizes the descriptive statistics for all the continuous and ordinal variables. On average, the fatalistic culture has the lowest mean value (*M* = 2.66). By contrast, the hierarchical culture has the highest mean value (*M* = 3.64), followed by individualistic (*M* = 3.54) and egalitarian cultures (*M* = 3.39). By sorting the organizational culture index of the respondents into the four dominant cultural types by identifying the culture on which they rated the highest, 36% of local governments are dominant hierarchy culture, 28% of them are dominant individualism, 26% of them are dominant egalitarianism, and 11% of them are dominant fatalism.

**Table 2:** Descriptive statistics.

**Variables**	**Observation (n)**	**Mean**	**Std. Dev**.	**Minimum**	**Maximum**
Dependent variables					
Equal public participation index	96	0	0.90	−1.82	2.38
Authentic public participation index	96	0	0.78	−1.54	1.59
Independent variables: organizational culture					
Fatalism	96	2.66	0.70	1.33	5
Egalitarianism	96	3.39	0.75	1	4.67
Hierarchy	96	3.64	0.57	2.33	5
Individualism	96	3.54	0.68	2	5
Control variables					
Median Income	96	89, 262.55	67, 462.47	34, 273	618,587
PerWhite	96	80.18	19.15	14	99
PerBachelor	96	40.60	22.60	6	89
Population	96	21,286.94	26,561.53	2,037	197, 899
Education	92	2.619565	0.8233194	1	4
Work Experience	92	4.076087	1.376679	1	5

*Median Income, Median household income; PerWhite, Percentage of whites only; PerBachelor, Percentage of people age 25 and older who hold a bachelor's or higher degree; Years Of Work, Years of working in government*.

**Figure 2:**
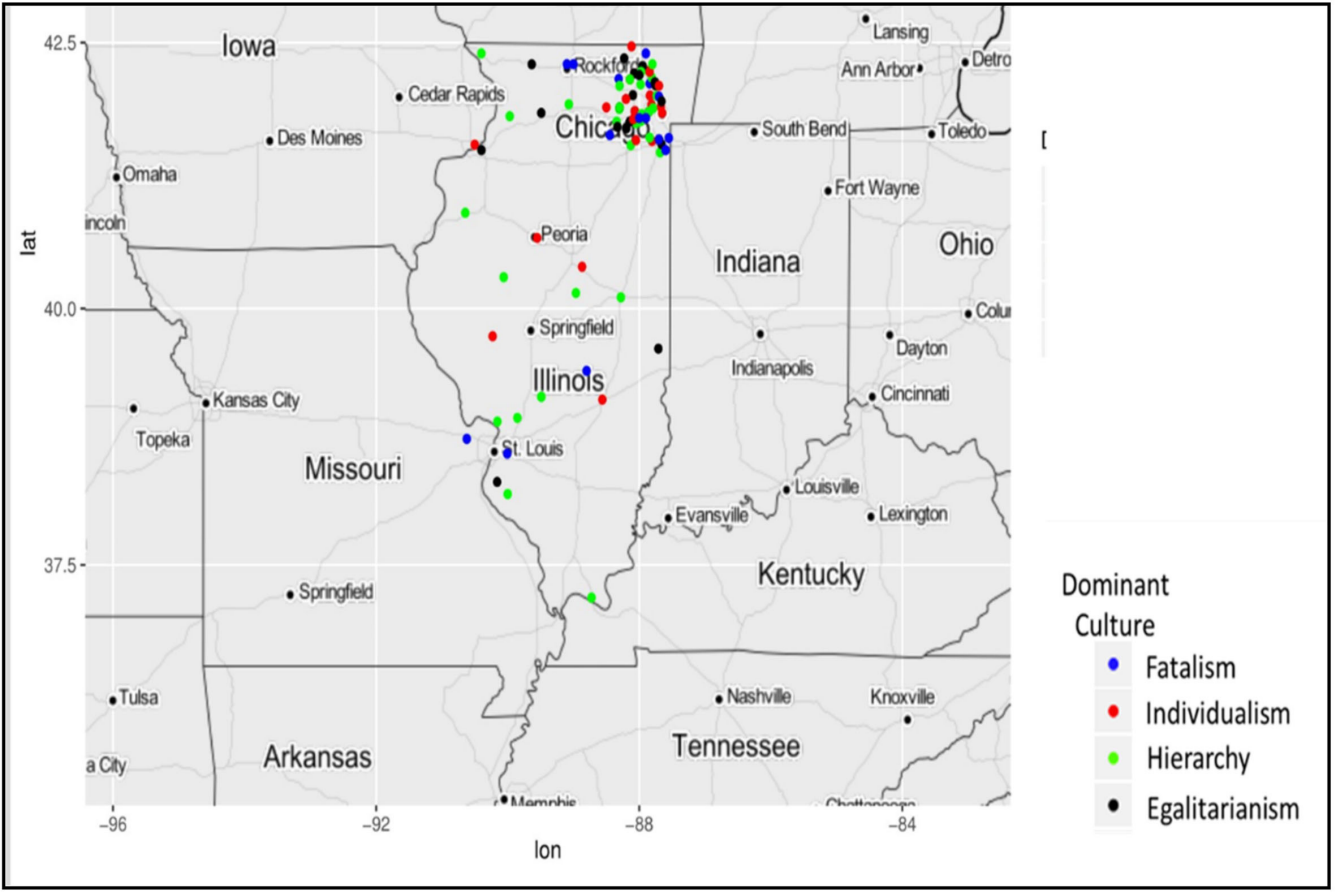
Mapping dominant organizational cultures.

As presented in Figure 2, most municipalities in this study are clustered around Cook County and five collar counties that border Chicago's Cook County, including DuPage, Kane, Lake, McHenry, and Will. Although the cities in the sample from the other regions of Illinois are far fewer, Figure 2 still shows some diversities of political cultures in every region of Illinois. Many years since, Frank et al. (1989) suggested that regionally based subcultures no longer exist in Illinois, culture and associated views may still vary widely throughout the state. Among the 21 municipalities from Cook County, three are fatalistic (Lynwood, Northbrook, and La Grange), eight are individualistic (Willow Springs, Western Springs, Winnetka, Bartlett, Des Plaines, South Barrington, Tinley Park, and Park Ridge), six are egalitarian (Streamwood, Homewood, Glencoe, Buffalo Grove, Broadview, Wilmette, and Northfield), and four are hierarchical (Justice, Barrington Hills, Park Forest, and Oak Park).

**Figure 3:**
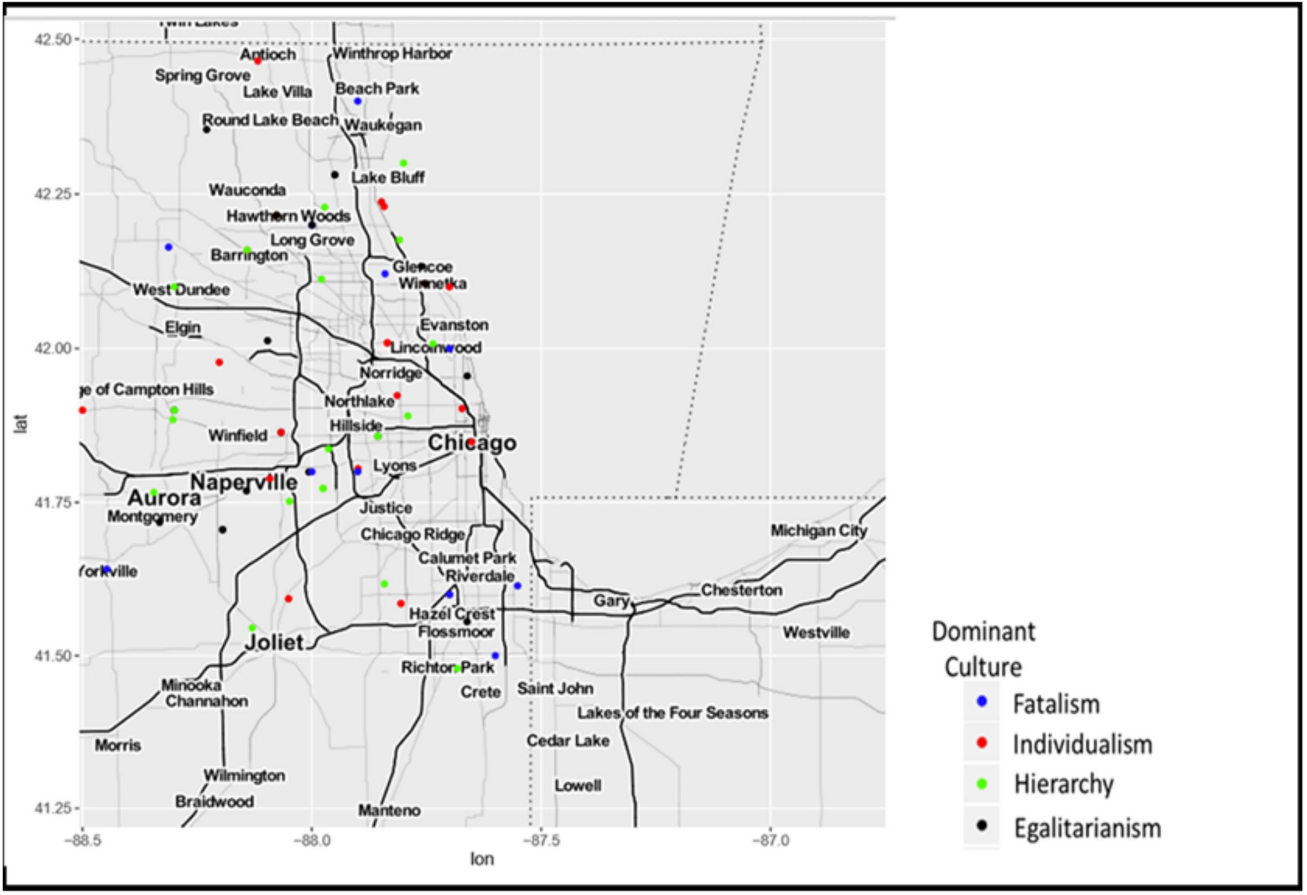
Mapping dominant organizational cultures around Cook County.

In the Collar counties that historically tilted Republican, the municipalities were culturally divided. Figure 3 zooms into the areas around Cook County. Woodridge, Westmont, Long Grove, West Dundee, Westmont, Batavia, Highland Park, Kildeer, Aurora, Cary, and Oak Brook have a hierarchical culture that values order. Among the individualist municipalities in collar counties, Elburn, Lisle, Antioch, Lockport, Lake Forest, Lake Zurich, and Villa Park have an individualist culture that values freedom. Beach Park, Glen Ellyn, Downers Grove, Lombard, Libertyville, Lakemoor, Naperville, Beecher, and Clarendon Hills have an egalitarian culture that values equality. Finally, Algonquin and Wadsworth have a fatalist culture.

The estimated item parameters in the IRT GRM are reported in Table 3. As shown in Table 3, two survey items or public participation strategies in the dimension of authentic participation consulting with (*a* = 2.64) and delegating direct power (*a* = 2.37) to the public have higher discrimination parameters than co-governing with the public (*a* = 1.86). Among the three survey items in the dimension of equal public participation, recruiting underrepresented groups has a higher discrimination parameter (*a* = 2.08) than recruiting lay citizens (*a* = 0.91) and randomly selected participants (*a* = 0.81). Columns of b1 to b4 in Table 3 reported the estimated thresholds of difficulty parameters. Because the item in procedural justice has five response categories, the GRM estimates the four threshold parameters for each item. In general, as the frequencies of adopting each of the public participation strategies increase, the difficulties in adopting these strategies increase. By comparing the difficulty parameter of b4, Table 4 shows that, in the dimension of equal participation, always recruiting randomly selected participants is a much more difficult strategy (*b*4 = 5.97) than recruiting lay citizens (*b*4 = 1.79) and underrepresented groups (*b*4 = 1.20); in the dimension of authentic participation, always delegating direct power (*b*4 = 2.15) is more difficult than co-governing (*b*4 = 1.98) and consulting (*b*4 = 1.94) with the public.

**Table 3:** Estimated item parameters of procedural justice.

	**Discrimination**	**Difficulty**	**b2**	**b3**	**b4**
	**parameter (a)**	**parameter (b1)**			
*Equal public participation*					
Lay citizens	0.91	−2.65	−1.35	0.02	1.79
Randomly selected participants	0.81	−0.77	1.15	2.23	5.97
Underrepresented groups	2.08	−0.94	0.30	0.94	1.20
*Authentic public participation*					
Consulting	2.63	−1.25	−0.36	0.43	1.94
Cogoverning	1.86	−1.48	−0.22	0.51	1.98
Direct Power	2.37	−1.04	0.10	0.87	2.15

**Table 4:** Pairwise comparisons using Tukey's test.

**Dominant organizational cultures**		**Equal public participation**		**Authentic public participation**	
**(I)**	**(J)**	**Mean Difference (I–J)**	**Std. Error**	**Mean Difference (I–J)**	**Std. Error**
Fatalism	Individualism	−0.66	0.37	−0.61	0.31
	Hierarchy	−0.82	0.36	−0.42	0.30
	Egalitarianism	−1.13^**^	0.38	−0.94^**^	0.31
Individualism	Fatalism	0.66	0.37	0.61	0.31
	Hierarchy	−0.16	0.26	0.19	0.21
	Egalitarianism	−0.47	0.28	−0.33	0.23
Hierarchy	Fatalism	0.82	0.36	0.42	0.30
	Individualism	0.16	0.26	−0.19	0.21
	Egalitarianism	−0.31^*^	0.27	−0.52	0.22
Egalitarianism	Fatalism	1.13^**^	0.38	0.94^**^	0.31
	Individualism	0.47	0.28	0.33	0.23
	Hierarchy	0.31^*^	0.27	0.52	0.22

** *p < 0.05*,

* *p < 0.1*.

**Figure 4:**
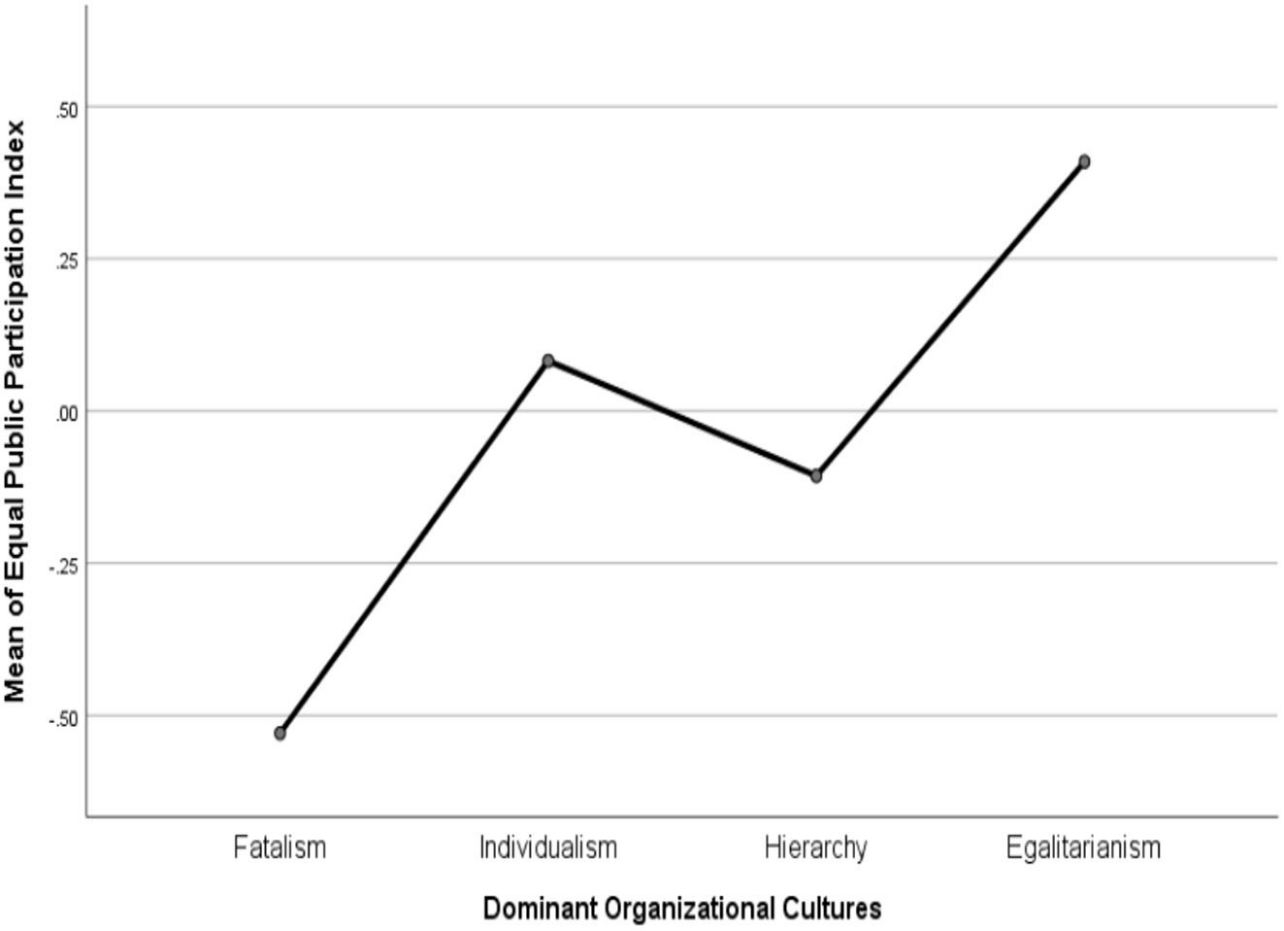
Means plots on equal public participation.

**Figure 5:**
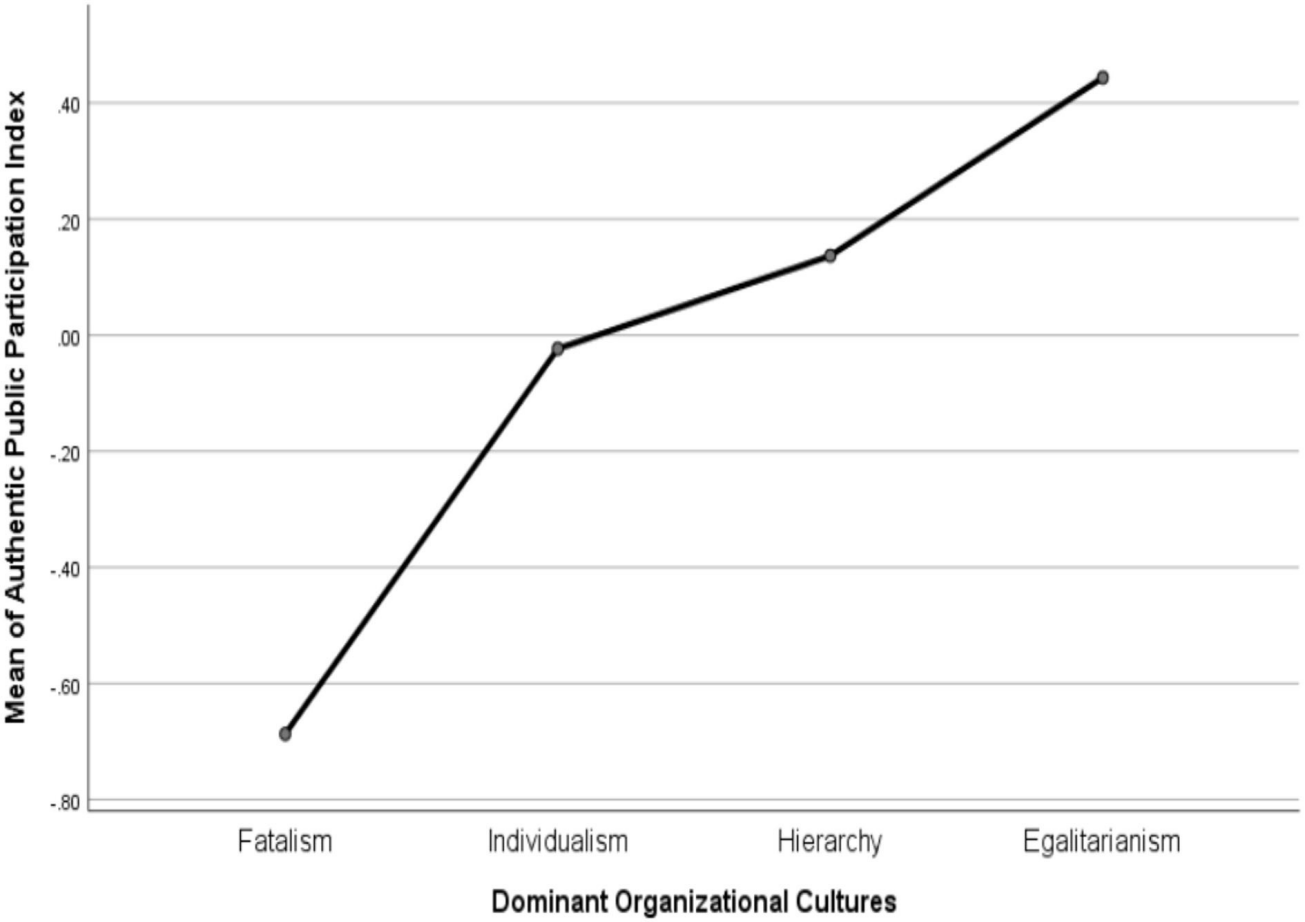
Means plots on authentic public participation.

The ANOVA test indicated that the mean of equal and authentic public participation is different across dominant organizational cultures at a 0.5 significance level. As reported in Figures 4, 5, organizational culture moves from fatalism to egalitarianism, both mean of equal and authentic public participation become higher. Local governments with a hierarchical or an individualist organizational culture have a middle level of procedural justice but individualist culture seems to be higher in equal public participation than do hierarchical governments. Tukey's method was used for *post-hoc* multiple pairwise comparisons (see Table 4). Specifically, compared to those with a dominant fatalist culture, local governments with a dominant egalitarian culture has a higher level of equal public participation [Mean Difference (MD) = 0.94, *p* < 0.05)] and authentic public participation (MD = 1.13, *p* < 0.05). Compared to those with a dominant hierarchical culture, local governments with a dominant egalitarian culture has a higher level of equal public participation but only at a marginal significance level (MD = 0.52, *p* < 0.1) and have no difference in authentic public participation. Moreover, no significant differences have been found between dominant individualist and egalitarian cultures.

### Step 2: Testing of Hypotheses in Multivariate Analyses

Table 5 presents the findings of this study on the relationship between the organizational cultural index and equal public participation, and Table 6 presents the relationship between the organizational cultural index and authentic public participation. However, before using the ordinary least squares regression method for multivariate analyses, this research first tests the bivariate association between the four cultural indices and two procedural justice indices, equal and authentic public participation, to make sure that the research on this field is on the right track. From Tables 5, 6, some of the precise associations that hypothesized occur in bivariate regressions: egalitarianism positively associate with both equal and authentic public participation, while fatalism negatively associates, and individualism positively associates with equal public participation. However, contrary to the author's expectations, individualism has no correlation with authentic public participation. Moreover, the hierarchy has no correlation with both equal and authentic public participation. As mentioned earlier, the four cultural indices should be tested separately in multivariate regressions to prevent multicollinearity. Therefore, there is no necessity to run multivariate regressions for cultures (e.g., hierarchy) that have been found no influence on procedural justice in bivariate analysis.

**Table 5:** Organizational culture and equal public participation.

	**Ordinary least squares regression**						
**Explanatory variables**	**Bivariate REGRESSION**				**Multivariate regression**		
	**Coefficient (Std. Error)**				**Coefficient (Std. Error)**		
*Cultural Indices*							
Fatalism	−0.32^***^ (0.10)				– −0.25^**^ (0.12)		
Individualism		0.30^***^ (0.10)				0.28^**^ (0.11)	
Hierarchy			0.01 (0.14)				
Egalitarianism				0.35^***^ (0.09)			0.31^***^ (0.10)
*Municipal Factors*							
Median Income					−0.04 (0.05)	−0.03 (0.05)	−0.05 (0.04)
PerBachelor					0.09 (0.08)	0.08 (0.08)	0.08 (0.07)
PerWhite					−0.13 (0.08)	−0.15^*^ (0.08)	−0.16^**^ (0.08)
Population					−0.03 (0.04)	0.01 (0.05)	−0.04 (0.04)
*Demographic Factors*							
Gender (Male)					0.00 (0.20)	0.06 (0.20)	−0.00 (0.20)
Race (Whites)					0.61^*^ (0.31)	0.77^***^ (0.25)	0.85^***^ (0.27)
Ideology					−0.13^*^ (0.07)	−0.12^*^ (0.07)	−0.09 (0.06)
Education					0.01 (0.12)	0.03 (0.12)	0.05 (0.13)
Work Experience					−0.02 (0.07)	−0.05 (0.07)	−0.04 (0.07)
Constant	0.84^***^ (0.28)	−1.08^**^ (0.37)	−0.05 (0.52)	−1.18^***^ (0.31)	0.51 (0.66)	−1.32^**^ (0.63)	−1.60^**^ (0.56)
R^2^	0.08	0.07	0.00	0.11	0.19	0.20	0.22
F-statistic	9.93^***^	8.60^***^	0.01	14.08^***^	2.82^***^	2.87^***^	3.36^***^

*** *p < 0.01*,

** *p < 0.05*,

* *p < 0.1 (two-tailed tests).*

*Median Income, Median household income; PerWhite, Percentage of whites-only; Per Bachelor, Percentage of people age 25 and older who hold a bachelor's or higher degree; Years Of Work, Years of working in government*.

**Table 6:** Organizational culture and authentic public participation.

	**Ordinary least squares regression**					
**Explanatory variables**	**Bivariate REGRESSION**				**Multivariate regression**	
	**Coefficient (Std. Error)**				**Coefficient (Std. Error)**	
*Cultural Indices*						
Fatalism	−0.35^***^ (0.12)				−0.30^**^ (0.15)	
Individualism		0.05 (0.16)				
Hierarchy			0.11 (0.17)			
Egalitarianism				0.42^***^ (0.10)		0.37^***^ (0.13)
*Municipal Factors*						
Median Income^a^					0.07 (0.06)	0.06 (0.06)
PerBachelor^b^					0.02 (0.09)	0.01 (0.09)
PerWhite^c^					−0.10 (0.09)	−0.14 (0.10)
Population					0.00 (0.05)	−0.01 (0.06)
*Demographic Factors*						
Gender (Male)					0.30 (0.22)	0.29 (0.22)
Race (Whites)					0.16 (0.34)	0.45 (0.38)
Ideology					−0.08 (0.11)	−0.03 (0.11)
Education					−0.00 (0.15)	0.05 (0.15)
Work Experience^d^					−0.06 (0.09)	−0.08 (0.09)
Constant	0.93^***^ (0.35)	−0.18 (0.58)	−0.39 (0.63)	−1.42^***^ (0.34)	1.04 (0.70)	−1.47 (0.79)
R^2^	0.08	0.00	0.00	0.12	0.14	0.17
F-statistic	8.59^***^	0.10	0.40	17.30^***^	2.32^**^	2.86^**^

*** *p < 0.01*,

** *p < 0.05*,

^*^p < 0.1 (two-tailed tests).

a *Median household income;*

b *Percentage of people age 25 and older who hold a bachelor's or higher degree;*

c *Percentage of whites only;*

d *Years of working in government*.

Our study is moved to the fully specified models to find whether the bivariate findings can withstand additional scrutiny. As shown in Table 5, all else being equal, a one-unit increase in a fatalist index leads to a 0.25 decrease in equal public participation, a one-unit increase in an individualist index leads to a 0.28 increase in equal public participation, and a one-unit increase in an egalitarian index leads to a 0.31 increase in equal public participation. As shown in Table 6, all else being equal, a one-unit increase in a fatalist index leads to a 0.30 decrease in authentic public participation, and a one-unit increase in an egalitarian index leads to a 0.37 increase in authentic public participation.

These may not seem like substantively important increases or decreases in the perception of local public administrators on equal and authentic public participation, but when one considers the full range of equal public participation, in this study ranges from −1.82 to 2.38, there may be a potential to think of the increase of, for example, 0.31 as more than a 7% increase in the perception of public administrators on equal public participation in environmental policy processes. Similarly, the full range of the authentic public participation in this study ranges from −1.54 to 1.59, there may be a potential to think of the increase of, for example, 0.37 as more than an 11% increase in the perception of public administrators on authentic public participation in environmental policy processes.

Among control variables including municipal and demographic factors in Table 5, the percentage of people with bachelor's degree or above, median household income, size of the population, public administrators' gender, educational level, and years of working experience in local governments do not make any difference in equal public participation. However, the percentage of whites in the municipality and race and ideology of public administrators can influence equal public participation. Compared to other races, local governments with public administrators who are whites have a higher level of equal public participation across all multivariate regressions in Table 5. Moreover, the percentage of whites and the ideology of public administrators have been found to possess correlations with equal public participation but these effects are not consistent across all multivariate regressions. An increase in the percentage of whites is negatively associated with equal public participation, which supports the literature on racial diversity, as communities with diverse ethnicities and races are correlated with strong social capital and high trust in the community (Graham et al., 2016) that fosters the recognition and participation of the disadvantaged. Moving from strongly liberal to conservative is negatively associated with equal public participation. For authentic public participation, as shown in Table 6, none of the control variables have correlations with authentic participation across all multivariate regressions. Therefore, it is suggested that the multivariate regressions corroborate the overall value of organizational culture for procedural justice.

## Discussion

Procedural justice is important not only because it is a public right in democracies but also as a process through which unequal distribution of environmental hazards and amenities can be addressed. Public participation plays a central role in procedural justice (Young, 2002). However, procedural justice is distinguished from traditional public participation by its emphasis on equal and authentic participation. Without recognizing the voice of and shifting the power to the socially and politically marginalized groups in the environmental policy-making process, policy decisions will still most likely generate disproportionate benefits for advantaged and privileged groups.

This study expands the empirical literature on the procedural justice environmental policy initiated by local governments. This research explains how procedural justice is manifested in two dimensions: equal and authentic public participation. Specifically, in IRT GRM, distinctive public participation strategies for improving equal and authentic participation show variations difficulties and frequencies of adoption across local governments.

While the role of organizational culture of local governments in explaining political participation has been studied previously (e.g., Sharkansky, 1969; Johnson, 1976; Dran et al., 1991; Morgan and Watson, 1991; Yang, 2005), the use of CT to characterize and study the influence of organizational culture on procedural justice in local environmental policy processes is new. Yet, CT has allowed the author to capture the cultural components that reflect a variation in the quality of network governance, which resides in high cultural bonds and the absence of the strong directives and status differences of the high grid. That is, high-quality network governance is promoted by an egalitarian organizational culture. Being able to specify this is especially important, as network governance is a key to promote public participation but is often operationalized on structural rather than cultural characteristics in existing research (Yang, 2005). Moreover, this research is the first research providing a full picture of four types of organizational cultures in explaining the procedural justice efforts of local governments in environmental policy processes.

This research furnished the two forms of evidence in support that organizational culture explains equal and authentic public participation much better than municipal characteristics, such as municipal socioeconomic conditions, and demographics of public administrators. The first was the polarized levels of equal and authentic public participation between local governments with a dominant fatalist and dominant egalitarian organizational culture. Because of its core value of equality, local governments dominated by an egalitarian culture support the normative values in procedural justice, equal, and authentic participation, at the highest level. Fatalism is least likely to include the general public and disadvantaged groups and shift the power to the public in a policy process, probably because of the apathy and cynicism about collective actions in this culture.

The second form of evidence, multivariate regressions, identified a mechanism that how these differences in the four types of dominant organizational culture are formulated from the four cultural indices: egalitarianism, individualism, hierarchy, and fatalism. As expected, an increase in egalitarianism showed an increase in both equal and authentic public participation in environmental policy processes, whereas an increase in fatalism showed a decrease in both equal and authentic public participation. These results are consistent with previous evidence that egalitarian public sectors are more committed to trust the general public and engage them in a policy-making process (Conner et al., 2016; Simmons, 2016). Some of the previous research defined fatalism as deliberatively designed randomness (Hood, 1998) that can increase the possibility that each member of the targeted population has an equal chance of being selected in public management (Stoker, 2002). However, this research found evidence that fatalism decreases equal public participation including randomly selected participants and supports other research in that fatalistic relations are characterized as the denial of the possibility of coordination or collective action (Ripberger et al., 2014; Swedlow, 2014). This result further suggests that local governments with fatalism are hesitating to delegate the power to the general public because they are reluctant to abandon their core values to achieve political equality. While having no influence on authentic public participation, individualism provides some spaces for procedural justice by increasing equal public participation. This finding is consistent with the theoretical assumption that individualists value freedom and may support pluralist democracy with checks and balances to maximize individual freedom (Verweij et al., 2011). However, because individualists suggest that public participation should be on a voluntary basis (Ney and Verweij, 2014), they are indifferent in the approaches to delegate the power to the public as long as the outcome is made from a competitive or bargaining process, driven by self-interest. Contrary to the author's expectations, the hierarchy has no correlation with both equal and authentic public participation. This result is inconsistent with previous evidence that hierarchical culture prefers a science- or an expert-dominance approach of public participation (Ney and Verweij, 2014). This research suggests that hierarchical local governments have no specific preference for equal and authentic public participation in the environmental policy-making process. Moreover, there is another possibility that some hierarchical local governments are more intended to engage the public in a policy-making process while others are not. More specifically, Wildavsky (2006) distinguishes the inclusive from the exclusive social hierarchy. Inclusive hierarchs want to inculcate traditional morality but still help the less fortunate (Swedlow, 2008), which may be thought of as approximate to communitarians: both types value a hierarchical order but also value caring. Moreover, Schwartz (1992) analysis of value structure suggests that although equality and social justice are distinct values, equality, caring, and social justice are closely related values. Therefore, inclusive hierarchy is closely related to egalitarianism, perhaps overlapping, which might make a space for public participation. By contrast, exclusive social hierarchs leave no room for public participation. A failure to distinguish these two types of hierarchical subcultures in this research may lead that the opposite effects of two hierarchical subcultures on procedural justice are offset.

In this study, municipal factors such as racial diversities measured by the percentage of whites and demographic factors of local public administrators can only explain, at best, the dimension of equal public participation. In all models, local governments with a city manager or chief administrative officer who is white and more liberal have a higher level of equal public participation. In most models, the percentage of whites decreases the level of equal public participation, which is consistent with previous evidence that racial diversity can broaden the participants and thus increase the representation of the public (Levine and Gershenson, 2014).

These findings have practical implications: first, public officials who want to improve the procedural justice in the environmental policy-making process should recognize that organizational culture, along with racial diversity of the community and race and ideology of public administrators, significantly shape the level of equal public participation. However, organizational culture is also important in influencing the decision of how much power is delegated to the public, while the effects of community and demographic factors are very limited. Thus, the extent of influencing the public can have in making final decisions, namely authentic public participation in this study, still lies with the government. Second, public officials should understand that procedural justice is not uniformly influenced by organizational cultures, depending on the dimensions of procedural justice, equal vs. authentic public participation. Finally, this research found that all four cultures have both similarities and differences in their effects on procedural justice. These findings imply that clumsy solutions generated by mixing all four modes (Verweij et al., 2011) may compensate for the drawbacks of each culture, generate a more pluralist public participation strategy, and improve procedural justice.

This research has many limitations. This study was based on the perceived culture and procedural justice of city managers or chief administrators, and future studies may want to use objective measures to validate the results. Future research should also consider the organizational culture at different levels of departments and perceived organizational culture from different staff within the same local government. Nevertheless, this study has merit for the case of two reasons. First, prior studies have successfully used the self-reported data of city managers or administrators for public participation research (Yang, 2005; Yang and Callahan, 2007; Smith-Walter, 2015). Second, related to the first limitation, some operationalizations of procedural justice components (e.g., randomly selected participants) may be too abstract to understand for the public administrators who took the survey and consequently may be interpreted differently by them. Failing to provide more concrete choices among public participation mechanisms could lead to inconsistent answers, which could threaten the internal validity of this study. Third, another limitation of this study is the small and unrepresentative sample, and future studies should make the research generalizable to other states. However, the generalizability of the findings in this study is enhanced because the surveyed local Illinois governments were diverse in population size and median household income levels and used the different forms of government. Finally, the Cronbach's alphas for a hierarchical culture is <0.5, which can lead to an unreliable measurement, the effect of measurement unreliability typically is to reduce the likelihood of rejecting the null hypothesis when statistical relationships are examined, a type two error (Swedlow and Wyckoff, 2009). Thus, the null hypothesis stating no relationship between hierarchy and environmental concern could be accepted inaccurately in this analysis. Future research should address the reliability problem by using techniques such as constructing more survey items for each culture.

## Data Availability Statement

The datasets presented in this study can be found in online repositories. The names of the repository/repositories and accession number(s) can be found below: https://doi.org/10.6084/m9.figshare.13193132.v1.

## Ethics Statement

The studies involving human participants were reviewed and approved by Northern Illinois University's Office of Research Compliance. The patients/participants provided their written informed consent to participate in this study.

## Author's Note

In EJ research, procedural justice is defined by equal and authentic public access to environmental policy-making processes. This research is the first research to specify and quantify specific strategies in these two dimensions of procedural justice. IRT GRM is used to show variations of difficulties and frequencies of adopting distinctive public participation strategies for improving procedural justice across local governments in Illinois, the USA. Moreover, this research introduces a new theory, culture theory, to measure and operationalize the perceived organization culture from public officials. By constructing an original survey, this research furnished two forms of evidence in support that perception of public officialorganizational culture can explain both equal and authentic public participation in procedural justice. Specifically, because of its core value of equality, local governments dominated by egalitarian culture support the normative values in procedural justice, equal and authentic participation, at the highest level. Fatalism is least likely to include the general public and disadvantaged groups and shift power to the public in the policy process. Individualism provides some spaces for procedural justice by increasing equal public participation.

## Author Contributions

The author confirms being the sole contributor of this work and has approved it for publication.

## Funding

This work was supported by Chongqing University Fundamental Research Funds for the Central Universities [grant number 2021CD8KXYGG006], National Natural Science Foundation of China (Grant No. 72104039), Humanity and Social Science Youth foundation of Ministry of Education of China (Grant No. 21XJC810001), and National Natural Science Foundation of China (Grant No. 72074035).

## Conflict of Interest

The author declares that the research was conducted in the absence of any commercial or financial relationships that could be construed as a potential conflict of interest.

## Publisher's Note

All claims expressed in this article are solely those of the authors and do not necessarily represent those of their affiliated organizations, or those of the publisher, the editors and the reviewers. Any product that may be evaluated in this article, or claim that may be made by its manufacturer, is not guaranteed or endorsed by the publisher.
