# Supplementary material for: How the Perception of Public Official on Organizational Culture Influences Procedural Justice in Environmental Policy Processes
Source: Front Psychol. 2021 Sep 27;12:626210. doi: 10.3389/fpsyg.2021.626210 (PMC8504256; doi:10.3389/fpsyg.2021.626210)
Supplement: Supplementary file 1 [file Data_Sheet_1.docx]

**Appendix: Survey Instrument**

**In this section, please answer the questions based on your experiences with the environmental policy processes in which you participated in the local government for which you currently work.**

Please rate the frequency with which your local government included the following participants.

- Lay, unpaid citizen stakeholders.
- Randomly selected participants.
- Selectively recruited participants from subgroups that are less likely to engage.

Please rate the frequency with which public participation...

- provided consultation and advice for sustainability plans.
- co-governed with local government to make sustainability plans and policies or strategies.
- exercised direct authority and power in sustainability planning.

Note: The order of questions will be randomized in this section. Response options are: 1. Never 2. Rarely 3. About Half the Time 4. Often 5. Always

**In this section, based on your experiences in your local government, how much do you agree or disagree with each of these statements? All questions pertain to the local government for which you currently work unless otherwise indicated.**

Hierarchy:

- The glue that holds us together consists of a sense of duty and respect for the distinct roles of each staff member.
- We emphasize clear lines of accountability.
- Not behaving according to one’s status or role in the organization is NOT tolerated.

Individualism:

- Our boss emphasizes that it is important to evaluate each employee’s performance individually.
- Promotions given on the basis of tenure alone are resented.
- Market values, such as price, cost, and profit, are the most frequently used terms in talking about our performance.

Egalitarianism:

- Our boss emphasizes consensus across all levels and units.
- Staff opinion is taken into account when promotions are given.
- We emphasize teams in which all members are equally important.

Fatalism:

- There is nothing holding us together and binding us to each other except for legal and managerial directives.
- We constantly have to react to things over which we have no control.
- It is difficult to know what our boss thinks is important.

Note: Response options are: 1. Disagree Strongly 2. Disagree 3. Neither Agree or Disagree 4. Agree 5. Agree Strongly

**Demographics and Background Questions**

How many years have you worked in government generally?

- Less than 1 year
- 1-2
- 3-5
- 5-8
- 8-10
- More than 10 years

Please indicate your race.

- White
- African-American
- American Indian or Alaska Native
- Asian-American
- Native Hawaiian or Other Pacific Islander
- Other
- Prefer not to say.

Please indicate your gender.

- Male
- Female
- Prefer not to say.

Please indicate the highest level of education you have received.

- Less than High School
- High School/GED
- Some College
- 2-year College Degree
- 4-year College Degree
- Master’s Degree
- Doctoral Degree
- Professional Degree (JD, MD)

When it comes to politics, do you think of yourself as:

- Strongly liberal
- Somewhat liberal
- Moderate or middle of the road
- Somewhat conservative
- Strongly conservative
